# Relevance of hazards in exoskeleton applications: a survey-based enquiry

**DOI:** 10.1186/s12984-023-01191-y

**Published:** 2023-05-31

**Authors:** Stefano Massardi, David Pinto-Fernandez, Jan Babič, Miha Dežman, Andrej Trošt, Victor Grosu, Dirk Lefeber, Carlos Rodriguez, Jule Bessler, Leendert Schaake, Gerdienke Prange-Lasonder, Jan F. Veneman, Diego Torricelli

**Affiliations:** 1 Neural Rehabilitation Group of the Spanish National Research Council (CSIC), Madrid, Spain; 2 Department of Mechanical and Industrial Engineering, University of Brescia (DIMI), Brescia, Italy; 3 Universidad Politécnica de Madrid, Madrid, Spain; 4 Laboratory for Neuromechanics and Biorobotics, Department for Automation, Biocybernetics, and Robotics, Jožef Stefan Institute, Ljubljana, Slovenia; 5 Department of Mechanical Engineering, Robotics & Multibody Mechanics Research Group (R&MM), and Flanders Make, Vrije Universiteit Brussel, Pleinlaan 2, 1050 Brussels, Belgium; 6 Department of Biomedical Signals and Systems, University of Twente, Enschede, The Netherlands; 7 Roessingh Research and Development, Enschede, The Netherlands; 8 Department of Biomechanical Engineering, University of Twente, Enschede, The Netherlands; 9 Hocoma Medical GmbH, Zurich, Switzerland

**Keywords:** Exoskeletons, Physical assistant robots, Wearable robots, Hazards, Hazardous events, Safety, Risk assessment

## Abstract

Exoskeletons are becoming the reference technology for assistance and augmentation of human motor functions in a wide range of application domains. Unfortunately, the exponential growth of this sector has not been accompanied by a rigorous risk assessment (RA) process, which is necessary to identify the major aspects concerning the safety and impact of this new technology on humans. This situation may seriously hamper the market uptake of new products. This paper presents the results of a survey that was circulated to understand how hazards are considered by exoskeleton users, from research and industry perspectives. Our analysis aimed to identify the perceived occurrence and the impact of a sample of generic hazards, as well as to collect suggestions and general opinions from the respondents that can serve as a reference for more targeted RA. Our results identified a list of relevant hazards for exoskeletons. Among them, misalignments and unintended device motion were perceived as key aspects for exoskeletons’ safety. This survey aims to represent a first attempt in recording overall feedback from the community and contribute to future RAs and the identification of better mitigation strategies in the field.

## Introduction

In the last two decades, exoskeletons have become a promising technology for the assistance, augmentation, and rehabilitation of healthy individuals and patients [1]. Recent research on exoskeletons has dramatically increased in the last years, as testified by the rapid growth of number of manufacturers in the global market [2]. However, this rapid inclusion of new solutions in the market has left behind safety-related standards and procedures, which are advancing at a slower pace. Performance, ergonomics, healthcare impacts, long-term safety, and other effects on humans have yet to be studied and understood, as testified by the lack of normative requirements on data collection and analysis in the current standards [3]. Risk assessment (RA) procedures must be carried out regardless of whether they are used as medical or non-medical devices. A standard RA normally starts determining which hazards in principle apply to a specific device. A hazard is a potential source of harm that in a determined scenario creates a so-called ‘hazardous situation’, i.e., a circumstance in which people are exposed to that or more hazards. Working with a device in a hazardous situation can lead to the occurrence of a ‘hazardous event’ (AE), resulting in harm for the user. Harm is a physical injury or damage to the health of people. The risk is generally calculated as a combination of the harm’s occurrence probability and its outcome’s severity. The purpose of a RA procedure is to identify each hazard (including hazardous situations and AEs) arising in all stages of the device’s life cycle, classify the severity and occurrence of the harms and estimate the risk of each identified hazard. The final step of this process is to judge whether the risk can be considered tolerable or not and, when not, to reduce the risk until it becomes tolerable [4].

Hazard analysis can start with brainstorming involving stakeholders from different fields, to provide a more comprehensive and multifaceted contribution. A RA for exoskeletons should be performed with the contribution of different and various actors to forecast the maximum number of possible AEs before properly assessing the risk level associated with each finding. The frequency rate of an event can be evaluated in pilot trials or stress tests that can vary from one device to another [5], whereas the occurrence of a known event per session can be assessed more rigorously [6, 7]. Severity remains a partially subjective metric that is not always clearly classified [5]. However, both occurrence and severity shall be classified according to the specific device evaluated.

We designed this survey aiming to address the most relevant events that can be part of an exoskeleton RA and asked the participants to rate their occurrence and severity according to their experience. The survey included a limited selection of AEs extracted from existing international standards and scientific literature that can be generally applied to a wide range of exoskeleton categories. Additionally, we asked the participant to select from a list of possible causes of the proposed events, according to their experience. This work is not meant to be a RA, as the participants are distributed over a wide range of devices, whereas a real RA shall be detailed for each device and its specific characteristics. This survey aims to collect impressions and opinions from experts in the wearable robotics field and analyze which aspects should be considered when performing a RA. The items composing the presented survey are extracted from a review of the main standards applicable to exoskeletons and related publications, as reported in the following section. The method section shows the construction and composition of the survey submitted to the community. The result section summarizes the responses collected. The discussion section includes the author’s comment on the results and the limitations of this work.

## Literature review

Standards and legislation are not meant to provide concrete case hazards and technical safety measures. Several existing European directives apply to exoskeletons depending on their application field. Machinery Directive [8] and the Medical Device Regulation (MDR) [9] are the principal European directives applicable for non-Medical and Medical applications respectively. They cover a wide range of devices and uses, as result, their content to address specific exoskeleton hazards is limited. Their purpose is not meant to guide users through a technical safety evaluation. However, compliance with harmonized standards automatically ensures compliance with EU directives. ISO EN 13482:2014 [10] covers the safety requirements for personal care robots. As a type C standard, it specifically addresses a type of machine and application, providing a more appropriate indication of possible hazards and safety issues. Exoskeletons used as physical assistant robots are part of the personal care robot family covered by this standard, where they are specifically classified as restrain-type physical assistant robots. ISO EN 13482:2014 lists many hazards related to the personal care domain, some of them being not (or partially) applicable to exoskeletons. From this standard we selected the most appropriate ones for this investigation:

- *Hazards related to charging battery* (clause 5.2): accidental contact with the charging connections present on the robots.
- *Hazards due to energy storage and supply* (clause 5.3): electrical parts other than the battery connections, uncontrolled release of stored energy in switching off/on the device, and any power failure of the device that could lead to an unexpected shutdown.
- *Hazards due to robot shape* (clause 5.6): contacts with robot physical parts such as sharp edges, corners, and surfaces that could lead to cuts, rubbings, and other related injuries.
- *Hazards due to emissions* (vibrations, clause 5.7.2): vibration emissions that could create discomfort and other effects on a user’s health (the standard also provides a range in Hz where vibrations shall be avoided).
- *Hazards due to stress, posture, and usage* (clause 5.9.2): physical stress and posture hazards due to a robot’s shape, weight, inertias, and other physical factors that constrain the user.
- *Hazards due to robot motion* (clause 5.10): mechanical instability (clause 5.10.2) that may produce any kind of intended or unintended motion. These hazards are related to the breaking or loosening of mechanical parts but also the instability of the attaching or removing procedure of the device from the user (clause 5.10.6). Clause 5.10 includes hazards concerning physical contact during human–robot interaction (clause 5.10.9), underlining how robot and its component shall be designed to reduce any interaction “as far as reasonably possible”. Interactions are composed of and influenced by a great variety of factors such as shears, frictions, forces and pressures, dynamic loads, and weights.
- *Hazards due to incorrect autonomous decisions and actions* (clause 5.12): wrong decisions and incorrect actions that might cause an unacceptable risk of harm from any personal care robot designed to make autonomous decisions and actions.

Recently, the technical report ISO/TR 23482:2020 [11] was published to support ISO 13482:2014. The document provides further guidance on the RA and risk reduction process to be conducted for a personal care robot. It contains examples of RAs for different types of personal care robots that can serve as an example for those users approaching ISO 13482:2014 to develop a RA. Clause 7.4 presents a partial RA example for a restrain-type physical assistance robot. The example includes five mechanical hazards and hazardous events related to unintended motion and unexpected control signals to the actuators, two electrical hazards related to battery and touching live connections, one thermal hazard concerning maintenance users, one ergonomic hazard related to discomfort, and one material hazard for the emission of dust. Although ISO 13482:2014 covers many uses of the exoskeleton as personal care robots, it doesn’t cover their application as medical devices. When considering medical devices, ISO EN 14971:2019 [12] is the reference document for the regulation of the RA procedure. This standard presents a list of hazards for generic medical devices, where exoskeletons can fit in electrical/mechanical associated hazards. However, the requirements for exoskeletons are different from other medical electrical equipment and medical electrical systems, since exoskeletons operate with a particular degree of autonomy and exchange energy with the patient in close contact and cooperation. ISO IEC 80601-2-78:2019 [13] includes particular requirements for basic safety and essential performance of medical robots for rehabilitation, assessment, compensation, or alleviation of lost body functions. It more specifically targets rehabilitation robots in medical applications and can be read as a hazard list for the addressed devices. This standard presents the following hazards applicable to exoskeletons:

- *Mechanical hazards associated with misalignment* (clause *201.9.101)*
- *Mechanical hazards associated with moving parts (clause 201.9.2)*
- *Unintended movement related to shared control between the patient, operator, or robot (clause 201.9.2.3.1.102)*
- *Movement beyond pre-set limits for individual patient movement (clause 201.9.2.3.101)*
- *Mechanical hazards associated with surfaces, corners, and edges (clause 201.9.3)*

Another document we consider is a Federal register from the Food and Drug Administration (FDA), which regulates medical devices in the United States having general applicability and legal effect. The register vol.80 n.36 [14] identifies nine risks associated with exoskeleton use, each of them combined with related special controls to mitigate the risk and provide assurance of safety and effectiveness. The identified risks are:

- Instability, falls, and associated injuries.
- Bruising, skin abrasion, pressure sores, soft tissue injury.
- Diastolic hypertension and changes in blood pressure, heart rate.
- Adverse tissue reaction.
- Premature battery failure.
- Interference with other electrical equipment/devices.
- Burns, electrical shock.
- Device malfunctioning resulting in unanticipated operation (e.g., device stoppage, unintended movement).
- Use error.

Clinical evaluation was also considered. Clinical studies often cover specific conditions of use but are not meant to analyze all the safety aspects of the device’s lifecycle. Some studies evaluated occurrences of AEs starting from the FDA’s list previously presented [5, 7, 15]. Based on this literature analysis, we selected and merged the most relevant items in a non-comprehensive list of general hazardous events that, in our opinion, are likely to be shared between exoskeleton devices. We excluded AEs like falls and collisions, normally investigated during in-field trials [16–18], being usually consequences of other primary AEs. Our list includes the following seven items:

- **Unintended/unexpected motion:** *Either a human or device fault leading to an undesired or unexpected motion.* An unintended motion is considered as a motion triggered by the user in an unintended way (i.e., a mistake in using the interface) while an unexpected motion also includes any device motions the user did not mean to trigger (i.e., device controller fault in the motion planning). We merged “unintended motion” and “unexpected motions” in a single item since the line between the two items is not always clear and easy to define. The item also includes excessive torques applied by the actuators and trajectory faults exceeding limits [16, 19, 20].
- **Unintended shutdown:** *Either a human or device fault leading to an unwanted or unexpected device shutdown.* Although unintended shutdowns can be thought of as part of unintended motions (previous item), they have been selected to form a singular item. This allows to specifically address all the situations where the device shuts down without causing unwanted motions.
- **Skin and soft tissue injury:** *Bruising, skin abrasion, pressure sores, soft tissue injury, primarily from the attachment points between the user’s body and the exoskeleton device.* Skin and soft tissue injuries are one of the most investigated AEs in the literature with a high rate of occurrence during exoskeleton use [6, 16, 20–25]. They can be consequences of many factors and cover several types of skin injury.
- **Misalignments:** *Offset between the exoskeleton and the human joints.* Misalignments can be considered a source of hazards since they produce higher forces at the interface, thus contributing to skin injuries, discomfort, and pain [16, 17, 26]. Misalignments are a very important factor to negotiate when using or designing exoskeletons [1, 27–30] thus, it has been included to highlight the experience users might have with them. The relation between misalignment and injury/discomfort is still largely unknown and requires attention.
- **Electrical fault:** *Battery failure, faulty cabling, and connectors, power shut down, discharges.* Electrical hazards are often divided into battery-related hazards and other types of accidental contacts, such as electrical malfunctioning [17, 19]. This single item encompasses all aspects of this family and reduces dispersion.
- **Hazardous vibrations:** *Vibrations making the motion difficult to control and/or uncomfortable.* Hazardous vibrations have been scarcely investigated in previous studies. Conversely, they have been mentioned as a source of hazards in the standards [10]. The item is then included to collect its relevance according to exoskeleton experts/users.
- **User error:** *User action or lack of user activity while using the device that leads to a different result than intended by the manufacturer.* User errors comprehend a wide family of possible hazards and events. For the sake of simplicity, they have been gathered in this single item.

## Methods

The survey was publicly announced and disseminated through the major mailing lists of the exoskeleton, human biomechanics and robotics communities. The survey was completely anonymous. Informed consent was presented at the beginning of the survey, where participants allowed responses to be recorded, analyzed and published. The survey was composed of 16 questions divided into three sections, plus an introductory section. In the introduction, participants are asked to select their professional/academic background and to provide a brief description of the exoskeleton that they used or operated (lower/upper limb, active/passive, rehabilitation/assistance robot, number of degrees of freedom of the device and the commercial name, if applicable).

### Section 1: frequency evaluation

In this section, the users were asked to evaluate how often they had to deal with each of the seven items presented. The following statement was proposed with a frequency scale composed of three levels [5]:

*Which of the following events have you experienced (or observed) during the use of exoskeletons? For each of them, please select how often you had to deal with it.*

- **Recurrent:** It happens regularly, from once per day to several times per session.
- **Occasional:** It happens occasionally, from several times per year to once per week.
- **Rare:** It happens rarely, from never to once per year*.*

At the end of the section, the users can add other remarkable events not mentioned in the list (and specify the corresponding frequency level) and any further comments they might have.

### Section 2: severity evaluation

The second section was designed to evaluate the severity level of each item proposed, following the same structure of the previous section. The following statement is proposed with a severity scale composed of three levels [5]:

*For each of the aforementioned events, select their experienced severity (see definition below). The focus is on the consequences on user health, e.g., potential injuries or adverse reactions. In case of more severe outcomes, select the most severe.*

- **Severe:** The event is incapacitating. It requires medical attention/treatment, and the use of the exoskeleton cannot be continued (e.g., bone fractures, skin lesions with complications).
- **Moderate:** The event interferes with the use of the exoskeleton but can be managed with simple measures. No prolonged effects (e.g., skin lesions without complications).
- **Minor:** The event is noticeable but easily tolerable. No medical intervention is needed and the use of the exoskeleton does not have to be interrupted, or only for a short rest (e.g., minor discomfort, reddening).

Once again, users could add other remarkable events not mentioned in the list (and specify the corresponding severity level) and any further comments they might have had.

### Section 3: Evaluation of causes

The last section investigates the potential causes related to each item and the possible dependencies between them. Possible causes are presented for each of them, being the result of the literature analysis and experience. The users could select more than one option and add other non-listed causes they have experienced.

#### Unintended shutdown

A. Proposed causes:
B. Electrical fault (malfunctioning, contacts).
C. Battery fault.
D. Loss of communication.
E. Insufficient durability of mechanical parts.
F. Use error (user’s unexpected or unintentional action).

#### Unintended/unexpected motion

A. Proposed causes:
B. Hazardous vibrations.
C. Electrical malfunctioning.
D. Sensor failure in reading.
E. Unintended triggers (Exoskeleton incorrectly reacting to body movements).
F. Mechanical instability/fault (slack stops, screws).
G. Use error (user’s unexpected or unintentional action).

#### Misalignments

A. Proposed causes:
B. Oversimplified kinematic structure of the exoskeleton.
C. Incorrect cuff positioning.
D. Cuff design (material, stiffness, number).
E. Insufficient durability (cuffs shift in extended uses).
F. Complex installation procedure.

#### Skin and soft tissue injury

A. Proposed causes:
B. Mechanical contacts (surfaces, edges, moving parts).
C. Burns, electrical shock (hot surfaces, electrical contacts).
D. Adverse interface design.
E. Adverse tissue reaction (biocompatibility).
F. Misalignment.
G. Kinematic limits exceeded (range of motion (ROM), velocity, force/torque).

#### Electrical fault

A. Proposed causes:
B. Cable break/fracture.
C. Precarious connections.
D. Contacts with body parts.
E. Short battery duration.
F. Other battery failures (overload, short circuit, heat).

#### Vibrations

A. Proposed causes:
B. Noisy signal from the controller.
C. Actuators (except the signal from the controller).
D. Resonances.
E. Cabling, connectors, faulty plugs.
F. Use error (user’s unexpected or unintentional action).

#### Use error

A. Proposed causes:
B. Wrong use of human-Exo interface (commands, menu, …).
C. Unforeseeable misuse (use not intended by the manufacturer).
D. Multiple commands at the same time.
E. Wrong settings.
F. Insufficient training.

The survey concludes with the option to add further AEs and/or causes along with any further feedback or input to this survey.

### Hazard’s relevance score

**Fig. 1:**
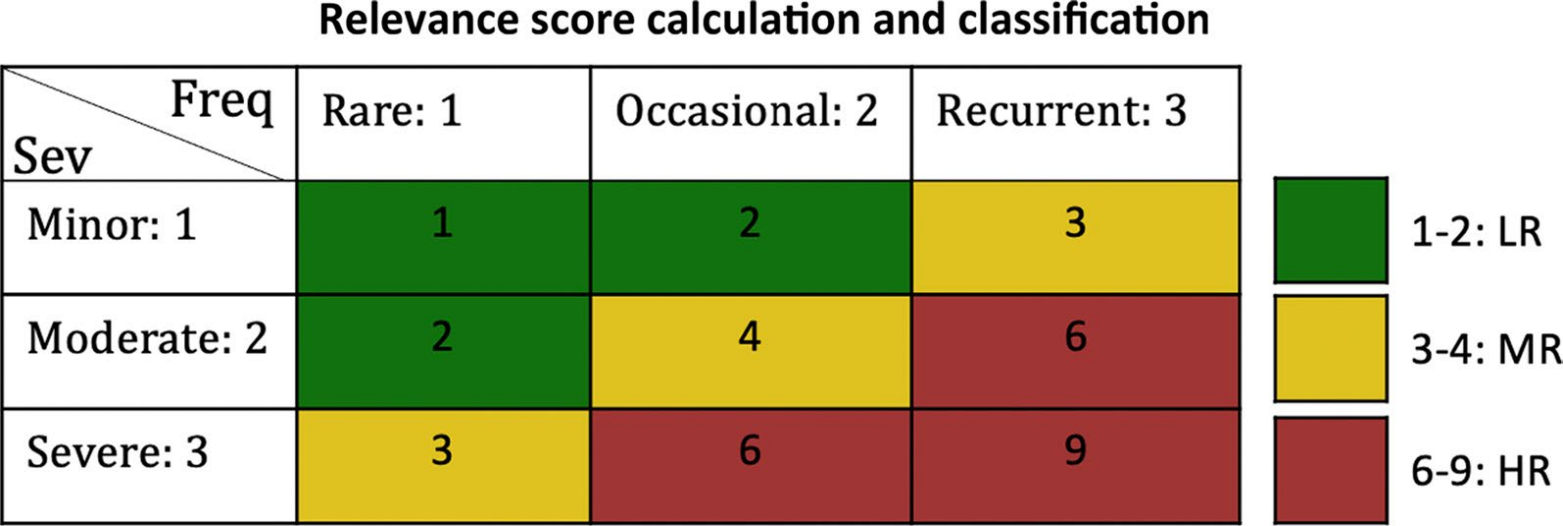
Relevance score calculation. The figure shows a table crossing frequency (Freq) values on the columns with severity (Sev) values on the rows. Scores are crossed and multiplied to get the relevance score of each item. Three combinations are considered: Resulting score from 1 to 2: low relevance (LR), Resulting score from 3 to 4: moderate relevance (MR). Resulting score from 6 to 9: high relevance (HR)

Hazards are evaluated in terms of the probability of occurrence and the severity of harm. Standards do not specify metrics to evaluate the probability and severity of harm, allowing organizations to select the method that is most suitable to them, either qualitative or quantitative [4, 10, 31]. Typical RA approaches use a risk matrix to indicate the level of acceptable risk by different combinations of severity and frequency levels [17, 19, 32, 33]. Developing a RA is out of the scope of this work and it cannot be conducted for such a wide range of devices and applications included by the respondents of this survey. However, inspired by RA procedures, we propose a composed score for each presented item, to create a priority list of which hazards are most considered/relevant for the community. As shown in Fig. 1 a score is given to severity and frequency options (minor/rare = 1, moderate/occasional = 2, severe/recurrent = 3). Each frequency score is matched with the corresponding severity given by the same participant. The combination results in 6 possible levels divided into three categories: (1–2) Low Relevance (LR), (3–4) Moderate Relevance (MR), and (6–9) High Relevance (HR). Although the proposed score is inspired by risk level evaluation in RAs, score levels in Fig. 1 are chosen arbitrarily, based on the authors’ experience and judgment. The proposed scores are not validated. They are used solely to get a rough estimation for comparisons between hazards and are not meant to represent any risk level.

## Results

**Fig. 2:**
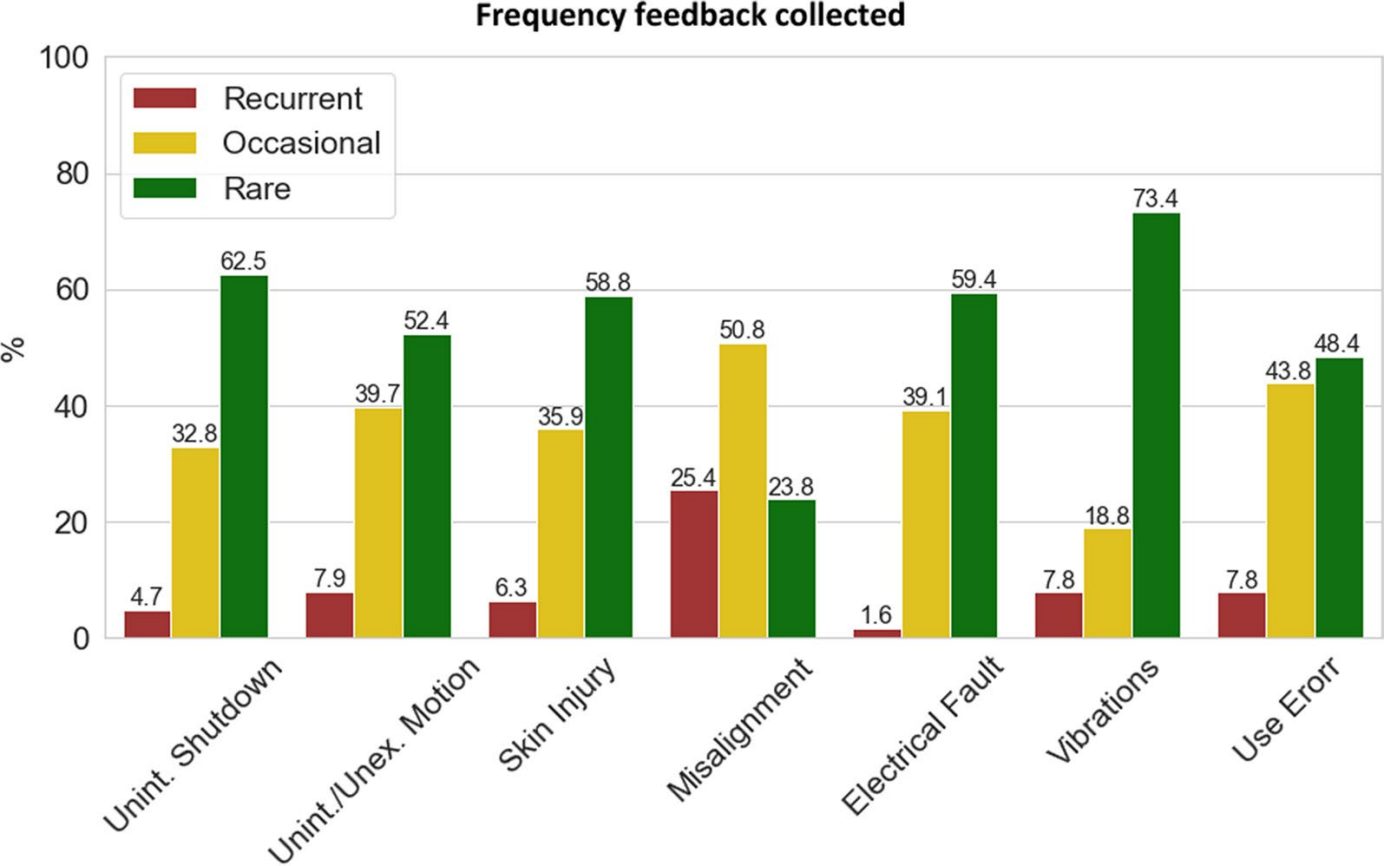
Frequency feedback. Results from Table 1 are presented in bar plot with the % of each frequency class for each AE. Red bars represent the % of respondents who recurrently experienced the event, yellow bars represent the % of respondents who occasionally experienced the event, and green bars represent the % of respondents who rarely experienced the event. Numbers on the bars refer to the exact % value recorded

**Fig. 3:**
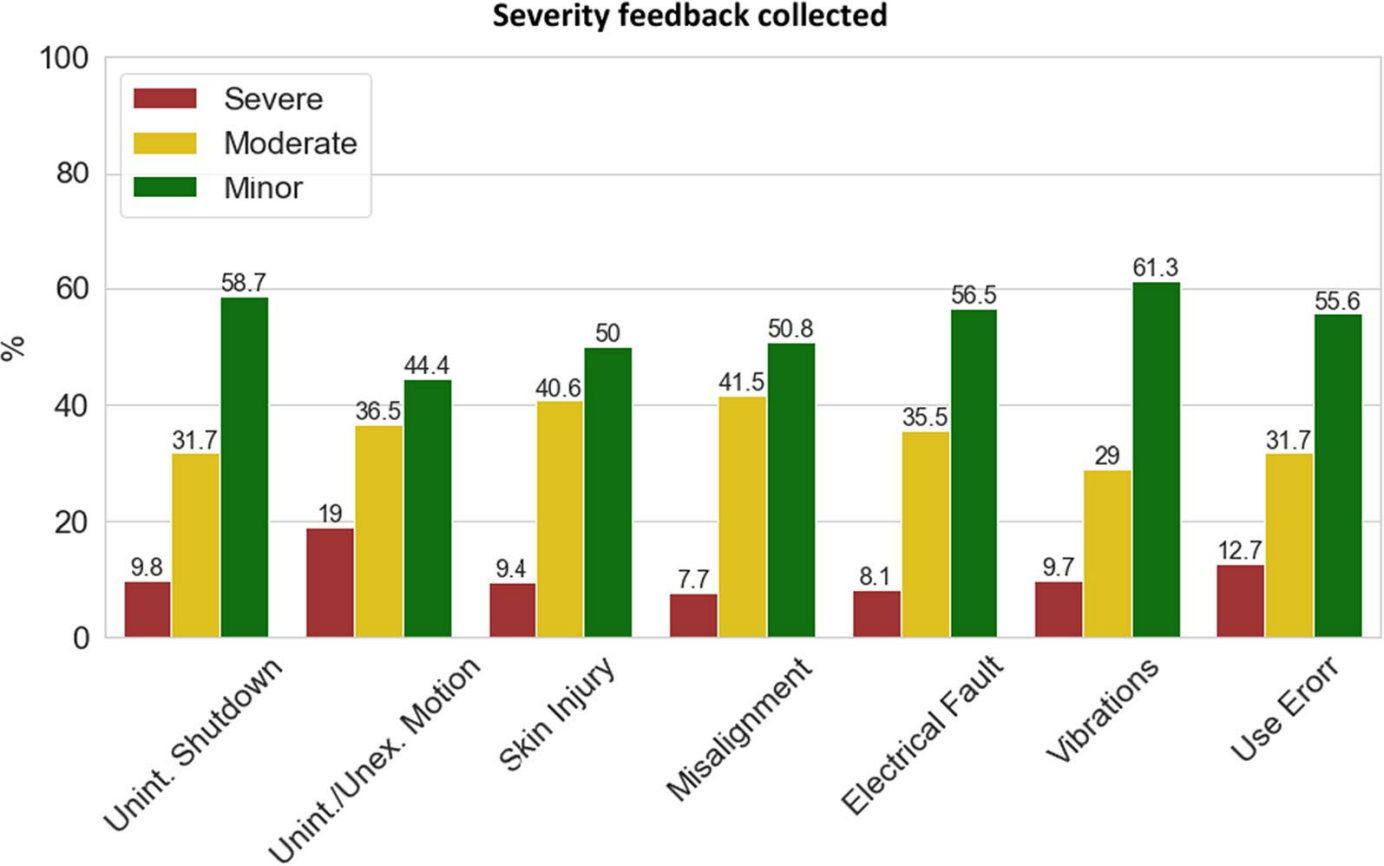
Severity feedback. Results from Table 2 are presented in bar plot with the % of each severity class for each AE. Red bars represent the % of respondents who experienced severe outcomes from the event, yellow bars represent the % of respondents who experienced moderate outcomes from the event, and green bars represent the % of respondents who experienced minor outcomes from the event. Numbers on the bars refer to the exact % value recorded

**Fig. 4:**
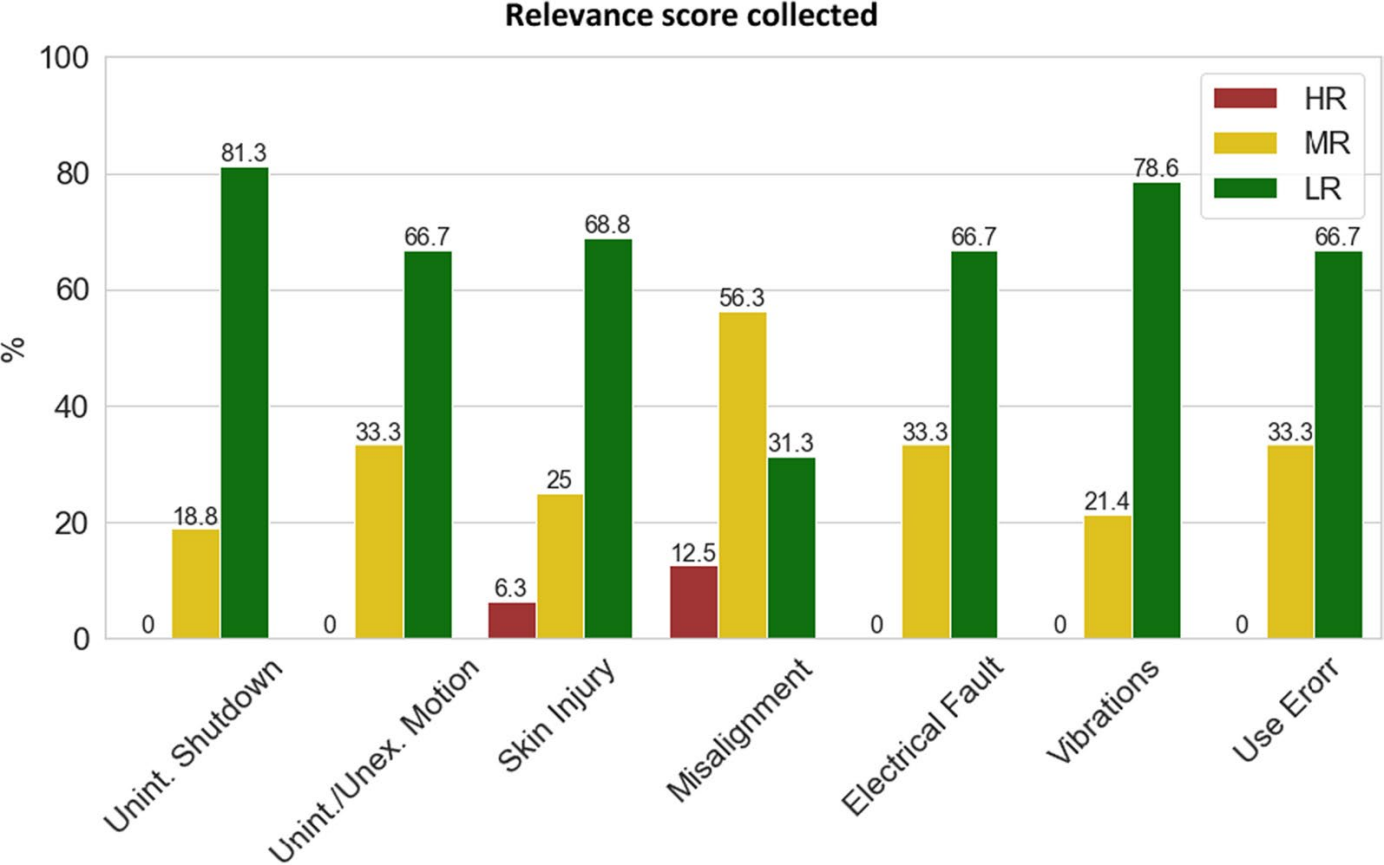
Resulting scores for each AE accordingly to results in Table 3 are presented in bar plot with the % of each resulting score associated with the answers from severity and frequency feedback. A resulting score from 1 to 2 is related to low relevance (LR), resulting score from 3 to 4 is related to moderate relevance (MR) and resulting score from 6 to 9 is related to high relevance (HR). Numbers on the bars refer to the exact % of the answers that are related with the relevance score

**Fig. 5:**
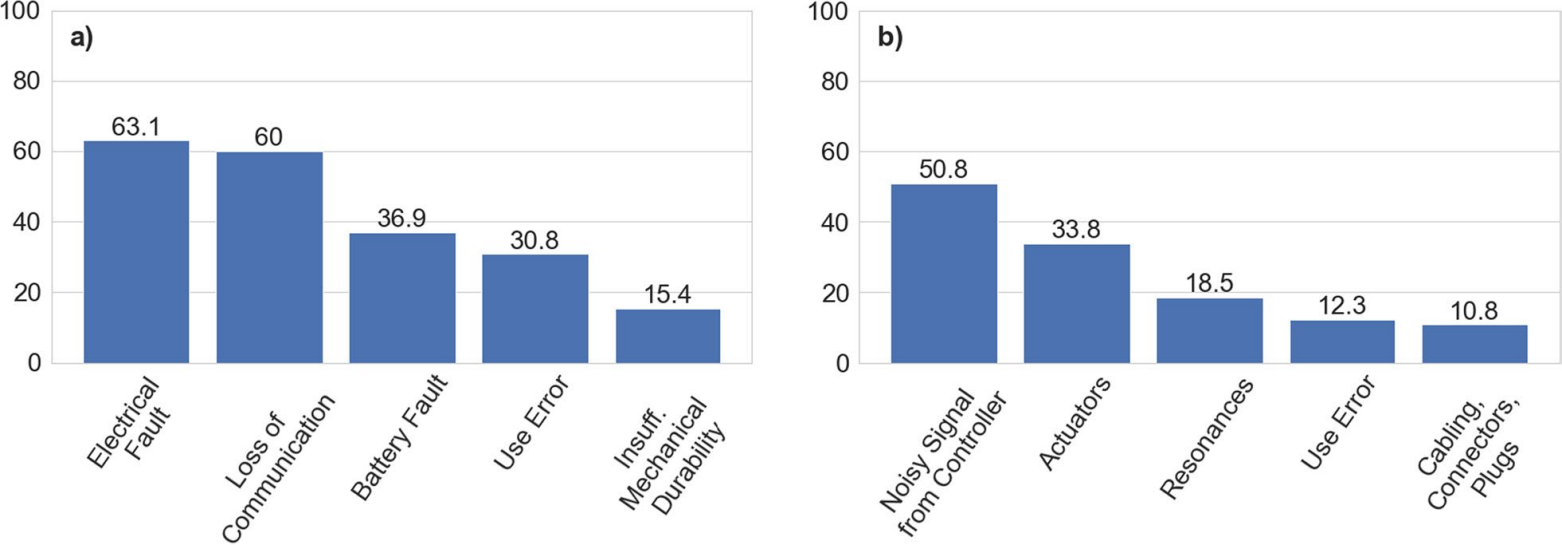
Experienced causing factors answered by participants, concerning unintended shutdowns (**a**) and vibrations (**b**). Numbers on the bars refer to the % of the answers collected for each cause

**Fig. 6:**
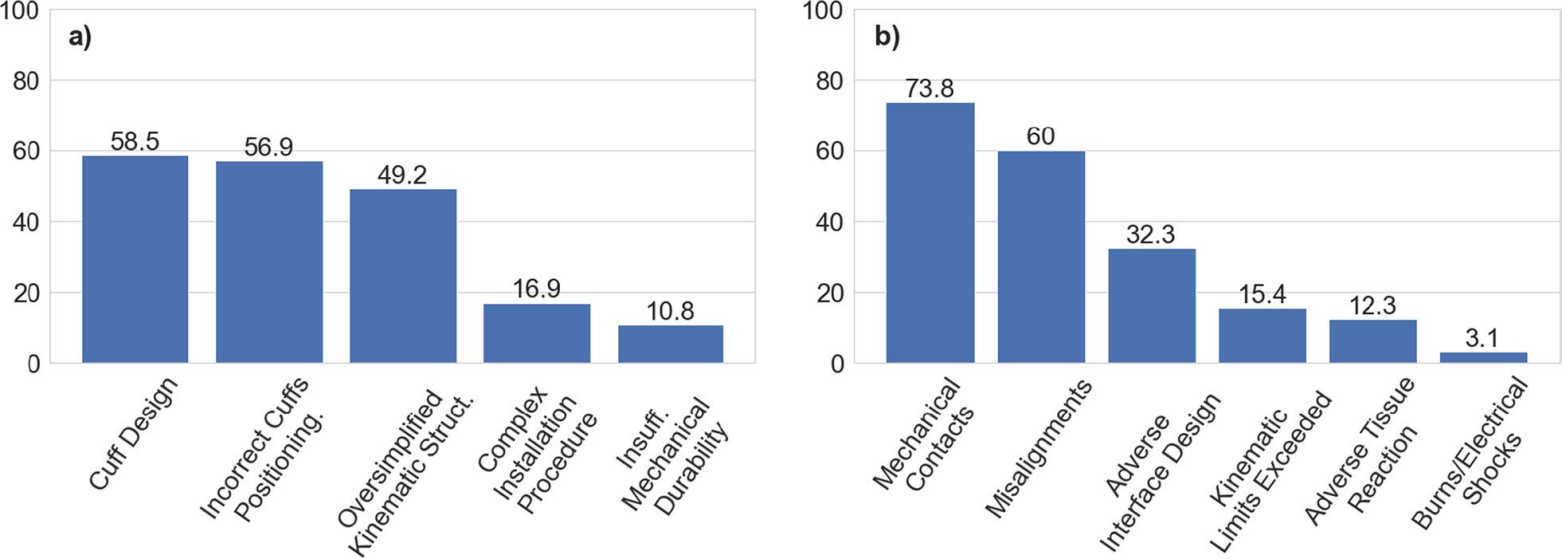
Experienced causing factors answered by participants, concerning misalignments (**a**) and skin and soft tissue injuries (**b**). Numbers on the bars refer to the % of the answers collected for each cause

**Fig. 7:**
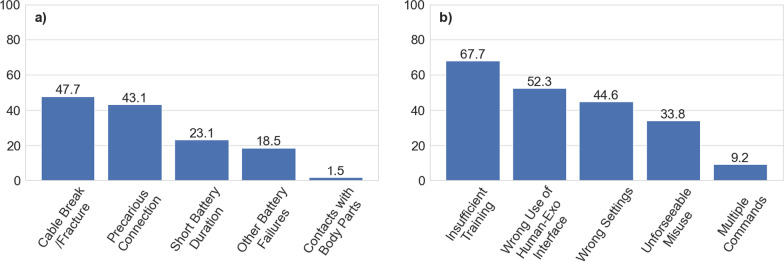
Experienced causing factors answered by participants concerning electrical faults **a** and use error (**b**). Numbers on the bars refer to the % of the answers collected for each cause

**Fig. 8:**
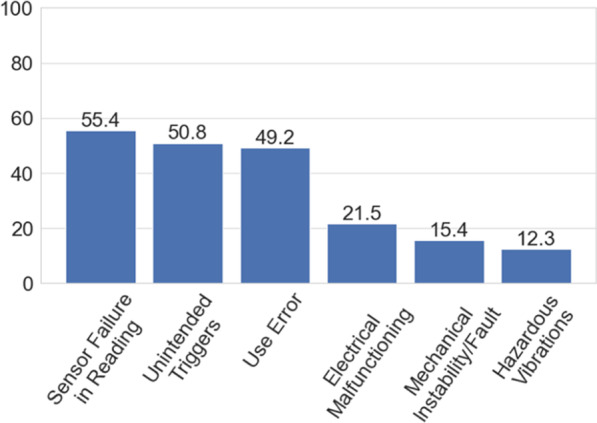
Experienced causing factors answered by participants concerning unexpected/unintended motion. Numbers on the bars refer to the % of the answers collected for each cause

The survey received 65 answers. 71% of the respondents (46 participants) worked in research fields, including 9 Ph.D. students (14%). 15 respondents (23%) were engineers from companies. The remaining 6% (4 respondents) were physiotherapists. Concerning the type of exoskeleton, the majority of respondents had experience with lower limb exoskeletons (59%), whereas 16 (25%) dealt with upper limb exoskeletons. 10 participants dealt with both upper limb and lower limb devices. One participant claimed not to directly work with exoskeletons. Nearly all participants (91%) dealt with active devices. 16 (25%) worked with passive devices and 11 with both active and passive devices. Concerning the field of use, 28 participants declared to deal with exoskeletons for rehabilitation (43%), 14 with assistive exoskeletons (22%), and 19 with assistive-rehabilitative devices (29%). Industrial field was represented by 7 participants (11%). One participant didn’t answer to the frequency evaluation of the events. The number of responses for each item is presented in Tables 1 and 2. Some items were not answered by the totality of the respondents, with a minimum of 62 answers in 2 items (Figs. 2, 3, 4, 5, 6, 7, 8).

**Table 1:** Frequency responses

Item	Recurrent	Occasional	Rare	Tot
Misalignments	16	32	15	63
Use Error	5	28	31	64
Unint./Unexp. Motion	5	25	33	63
Vibrations	5	12	47	64
Skin and soft tissue injuries	4	23	37	64
Unintended shutdown	3	21	40	64
Electrical fault	1	25	38	64

For each item the number of responses collected in the frequency section for “Recurrent”, “Occasional” and “Rare”. Column “Tot” is the total number of responses for each item

**Table 2:** Severity responses

Item	Severe	Moderate	Minor	Tot
Unintended/Unexpected motion	12	23	28	63
Use Error	8	20	35	63
Misalignments	5	37	33	65
Skin and soft tissue injuries	6	26	32	64
Unintended shutdown	6	20	37	63
Electrical fault	5	22	35	62
Vibrations	6	18	38	62

For each item the number of responses collected in the severity section for “Severe”, “Moderate”, and “Minor”. Column “Tot” is the total number of responses for each item

**Table 3:** Relevance score calculated responses

Item	LR Low relevance	MR Moderate relevance	HR High relevance	Tot
Misalignments	10	25	28	63
Unintended/Unexpected motion	6	19	37	62
Use Error	6	15	42	63
Skin and soft tissue injuries	4	17	42	64
Unintended shutdown	4	13	46	63
Vibrations	4	13	45	62
Electrical fault	0	18	44	62

For each item the number of responses matching scoring 1–2 (Low relevance), 3–4 (Moderate Relevance), 6–9 (High Relevance). The score is given by the product between frequency and severity score. Column “Tot” is the total number of responses for each item

Causes were investigated in the last survey section where participants could select from the proposed causes. Causes were the result of literature analysis and the author’s experience. The users could select more than one option and add other non-listed causes they might have experienced.

**Unintended shutdown:** Electrical fault 41 (63%), battery fault 24 (37%), loss of communication 39 (60%), insufficient durability of mechanical parts 10 (15.4%) and use error 20 (30.8%).

**Vibrations:** Noisy signal from the controller 33 (50.8%), actuators 22 (33.8%), resonances 12 (18.5%), cabling, connectors, faulty plugs 7 (10.8%) and use error 8 (12.3%).

**Misalignments:** Oversimplified kinematic structure of the exoskeleton 23 (49.2), incorrect cuffs positioning 37 (59.9%), cuff design 38 (58.5%), insufficient durability 7 (10.8%), and complex installation procedure 11 (16.9%).

**Skin and soft tissue injury:** Mechanical contacts 48 (73.8%), burns and electrical shocks 2 (3.1%), adverse interface design 21 (32.3%), adverse tissue reaction 8 (12.3%), misalignments 39 (60%) and kinematic limits exceeded 10 (15.4%).

**Electrical fault:** Cable break/fracture 31 (47.7%), precarious connections 28 (43.1%), contacts with body parts 1 (1.5%), short battery duration 15 (23.1%) and other battery failure 12 (18.5%).

**Use error:** Wrong use of human-Exo interface 34 (52.3%), unforeseeable misuse 22 (33.8%), multiple commands at the same time 6 (9.2%), wrong settings 29 (44.6%), and insufficient training 44 (67.7%).

**Unintended/Unexpected motion:** Hazardous vibrations 8 (12.3%), electrical malfunctioning 14 (21.5%), sensor failure in reading 36 (55.4%), unintended triggers 33 (50.8%), mechanical instability/fault 10 (15.4%) and use error 32 (49.2%).

## Discussion

The different backgrounds of the respondents hamper the achievement of specific conclusions and observations for a single device category. Respondents may have referred to one specific device but also to exoskeletons in general. We can still suppose that the result is an average, general estimate in the field, which cannot apply to any device and we shall avoid misunderstandings that might lead to read the results as a general RA. However, the result of this survey also represents a picture collected by an audience of real users operating outside the laboratory or clinical conditions. Such a result can favor a more concrete view of how exoskeletons are perceived, not only limited to research and scientific literature. The items presented as a list of hazards that usually/typically apply to exoskeletons can only be taken as a reference for RAs. Frequency and severity feedback shall be read with the knowledge of the variety of devices and conditions considered by the respondents, and cannot be related to an accepted frequency/severity classification of a specific RA.

The frequency analysis presented low variability among the responses. All the items, except misalignments, received a “rare” occurrence score from at least the 50% of the participants while “recurrent” was less than 10% of the answers. Severity evaluation also did not show significant trends with the consequences of the events averagely rated as “minor”. Misalignments were rated as “recurrent” by 25% of the respondents. A recurrent event was proposed as something happening from once a day to several times per session, meaning that misalignments are often daily problems for the users. Our definition for misalignments was “an offset between the human and the device joint”. This event is impossible to avoid considering that robot kinematics is just an approximation of the human body. Conversely, misalignments also received a 24% of “rare” occurrence rate. Misalignments might be considered negligible for some applications. One comment underlined how they were observed for paraplegic users (specifically taller users) and not for healthy users, suggesting that misalignments were noticed only for remarkable offsets. The controlled conditions from clinical environments might here influence the result since improper fitting and unprecise positioning are more easily avoided. None of the remaining events were frequently experienced with electrical malfunctioning collecting the lowest frequency score. This is understandable for devices in a commercial or pre-commercial stage since requirements for electrical safety are far clearer than for all the other event types. IEC-60601 is indeed very detailed on specific rules and safe limit values. This can perhaps be considered a simple field to comply with, in terms of knowing what can and needs to be done to make the system as (electrically) robust and safe as possible.

While the frequency evaluation might be based on a quantitative scale (one can record the occurrence rate of an event on a determined time unit), severity mostly relies on qualitative evaluations. The absence of clear and measurable criteria able to define severity makes the results more dependent on the specific device and application considered. Events leading to a severe injury are relatively rare in exoskeletons, for this reason, respondents might have less experience in rating severe AEs. In a recent review on exoskeleton risk management [16], two bone fractures were reported due to the occurrence of misalignments while the remaining listed events led to no injuries or skin damages that were resolved in a few days. Another review on AEs in stationary robots (e.g., Lokomat) collected 3 severe AEs out of 169 reported events although 43 remained unrated. However, the reported number of AEs could also be an underestimation, since reviews are limited to published reports. From the results, more than 10% of the participants experienced severe outcomes after unintended/unexpected motion (19%) and use error (12.7%). Unintended and unexpected motions can indeed lead to falls, having then higher hazardous potential compared to other hazards. The 19% of event’s consequences classified as severe can anyway raise a flag since “severe” was associated with incapacitating outcomes. From some collected comments, the participants pointed out how their severity score was relatively low because they were testing the device under the supervision of clinical staff, prevention measures, or under very limited and constrained conditions, which reduced the overall risk level. As we stated for misalignment, frequency scores are normally influenced by the presence of clinical staff supervision and controlled conditions. The fact that occurrence and severity are based on tests performed in a clinical environment may indeed affect the perception of the users in evaluating the events. Both severity and frequency can indeed be strongly affected by studies performed in presence of staff members monitoring the user and the system. What the community can communicate about their perceived experience in exoskeleton safety can be far from reality. This point is stressed by the poor knowledge that technical personnel may have about safety aspects. Additionally, safety tests without intervention are difficult to perform when they include humans. Part of the respondents might also have provided a more technical experience rather than a knowledge of medical or physical outcomes.

A lack of awareness from users and technicians might also explain the low severity score of the experienced outcomes. For example, the appearance of skin injuries can even occur days after exoskeleton use, and the user may not perceive the injury when doffing the device. In some cases, the supervisor might not pay attention to the harm the user is experiencing. Clinical studies normally focus on gait or task-related parameters without considering AEs, which can be of difficult detection. The combination of severity and frequency scores as indicated in Table 3 (and Fig. 4) produced a generally low relevance score for the proposed events. This can be interpreted as a matured confidence of the users towards these devices, but also as a lack of experience regarding the possible hazards and risks in these applications. Existing literature on exoskeleton safety is indeed much more limited when compared to the literature on electromechanical features, design, or control strategies. AEs are also poorly published, although investigators are obliged to list the ones occurring, with the obligation to take action in case of serious AEs. Further analysis of events’ causes showed some more recognizable trends. 60% of the participants identified electrical faults and loss of communications as causes for unintended shutdowns. However, electrical faults also received the lowest risk score in the evaluation, in contrast with the unintended shutdown risk results. Electrical faults were on their side mainly attributed to issues with cables and precarious connections, although the frequency of these events was one of the lowest recorded. As a consequence, unintended shutdowns also received a low-frequency score.

Skin damages were associated with mechanical contacts (73.8%) and misalignments (60%). This result shows how the design of the device plays a key role in defining safe shapes, surfaces, and attachment designs, which can otherwise lead to harmful and uncomfortable use. Misalignments can be related to the design phase of the device. Attachment strategies and materials directly influence the capability of the device to be well aligned and maintain the desired configuration. Misalignments were linked to three main aspects, namely cuff design, incorrect cuff positioning, and oversimplified kinematics. As previously said, the simplicity of the device could reduce the overall complexity but increase deviations from real human kinematics. Cuffs and interfaces shall be a developers’ priority to ensure safe human–machine contact and communication. However, incorrect cuff positioning could be also considered use error and not a design error (too high or low, rotated, etc.). More than 50% of the participants identified unintended triggers (exoskeleton incorrectly reacting to body movements) and sensor failure in reading as potential causes for unintended and unexpected motions, whereas still half of the participants also identified use errors as an important cause. If we analyze use errors, respondents highly agree on insufficient training as the major cause (67.7%). Training is one of the mitigation measures suggested by the FDA to decrease the level of risks. The other two major items for use errors are “wrong use of exoskeleton interface” and “wrong settings”, which might be also related to a lack of training and practice of the device, equally contributing to an AE occurrence. Together with training, user errors can be mitigated by improving usability testing where the user can provide useful feedback on the device’s design and user interface. From this point of view, pilot tests could be of great importance for a deep and practical knowledge of device safety.

## Limitations

The relatively limited number of respondents, together with the result’s imbalance in device type and participant’s background (especially between industry and academy) did not allow to extract separate conclusions from the different domains. Thus, no targeted analysis of the proposed events was possible.

Hazards are not all applicable in the same way to all devices. A generalization had to be applied considering that the same hazard can lead to one outcome in one device, in one situation, and a different outcome in another device in another situation. Due to this variability, this work can only claim to collect feedback on exoskeletons’ hazards and hazardous events in terms of occurrence, severity, and causes that the respondents have experienced. The background and experience of each participant can represent strong confounders for the analysis of the results. Attention shall be paid since the provided feedback can be driven by the participant’s perceived risk, which can sensitively change depending on multiple factors, such as the different experiences of AEs when dealing with exoskeletons prototypes vs commercial devices, or healthy users related events vs scenarios with patients.

The vast majority of respondents were researchers presumably working with exoskeletons in a very controlled environment. For this reason, analysis can be performed based on the authors’ expertise and best practice but not knowing whether the respondents would have rated it the same way in an actual RA. Further improvements would be to expand the survey to differentiate between real-world applications and clinical trials in a laboratory as well as differentiate between commercial devices and prototypes.

## Conclusions

This article is one of the first attempts to collect feedback from different fields and applications in the exoskeleton community. It represents an interesting point of view on how safety factors can be perceived by real users and experts in the sector.

The participants could answer about the relevance of exoskeletons hazards in terms of occurrence and severity outcomes as well as potential causes. The conducted survey collected user experiences and general considerations on the safety of these devices, highlighting relevant connections among the presented events and pointing out important characteristics that researchers and developers shall focus on. Misalignments were the most recurrent adverse event (AE) and were mainly linked to design issues. Nevertheless, a consolidated agreement on misalignment definition is still missing, which may have introduced data dispersion.

Unintended motion was on average rated as the most dangerous event and found to be due to sensors and human errors, such as training and understanding the device.

Overall, and somehow unexpectedly, the majority of AEs did not reach high severity and frequency ratings. However, these results cannot be taken as a real risk assessment (RA). Each manufacturer shall decide what combination of frequency and severity is acceptable for each specific device and its intended use. The items presented to the respondents and their results can only be taken as a reference for future RAs.

The use of exoskeletons outside clinical environments and without expert personnel is still limited. These controlled conditions can influence the perception of how the device can produce AEs. For this reason, developers shall also stress tests in scenarios as near as possible to the outside world conditions.

## Data Availability

Not applicable.

## Abbreviations

AE: Adverse event
HR: High relevance
LR: Low relevance
MR: Moderate relevance
RA: Risk assessment
